# Trauma-Informed Design of Supported Housing: A Scoping Review through the Lens of Neuroscience

**DOI:** 10.3390/ijerph192114279

**Published:** 2022-11-01

**Authors:** Ceridwen Owen, James Crane

**Affiliations:** 1 School of Architecture and Design, College of Sciences and Engineering, University of Tasmania, Launceston, TAS 7250, Australia; 2 Tasmanian School of Medicine, University of Tasmania, Hobart, TAS 7000, Australia

**Keywords:** trauma-informed design, neuroscience, architecture, domestic violence, homelessness, housing

## Abstract

There is growing recognition of the importance of the design of the built environment in supporting mental health. In this context, trauma-informed design has emerged as a new field of practice targeting the design of the built environment to support wellbeing and ameliorate the physical, psychological and emotional impacts of trauma and related pathologies such as Post Traumatic Stress Disorder (PTSD). With high levels of prevalence of PTSD among people escaping homelessness and domestic violence, a priority area is the identification and application of evidence-based design solutions for trauma-informed supported housing. This study sought to examine the scope of existing evidence on the relationship between trauma, housing and design and the correlation of this evidence with trauma-informed design principles, and to identify gaps and opportunities for future research. In response to the commonly articulated limitations of the evidence-base in built environment design research, we combined a scoping review of literature on trauma, housing and design with insights from neuroscience to focus and extend understanding of the opportunities of trauma-informed design. We found that while limited in scope, there is strong alignment between existing evidence and the principles of trauma-informed design. We also identify three areas of future research related to the key domains of safety and security; control; and enriched environments.

## 1. Introduction

Human health is inextricably tied to the design of the built environment. While the relationship between design and disease has a long history, more recently, the shift from pathogenesis (disease) to salutogenesis (wellness) has driven research investigating the positive relationships between design and wellbeing. Aligned with this shift is a burgeoning interest in the relationship between built environment design and mental health and wellbeing. This interest is accelerating as mental health is recognized as a major public health crisis.

One relatively new area of interest is the field of trauma-informed design, which targets the design of the built environment to support wellbeing and ameliorate the physical, psychological and emotional impacts of trauma. Traumatic-experiences can have a prolonged and debilitating negative impact on a person’s sense of self-worth, safety, and perceived control over the environment [1]. This is particularly the case for those who develop trauma-related pathologies, such as Post-Traumatic Stress Disorder (PTSD). With high levels of prevalence of PTSD among people escaping homelessness and domestic violence, a priority area is the identification and application of evidence-based design solutions for trauma-informed supported housing.

With scant literature in this area, we sought to undertake a scoping review to identify the existing evidence, and to interrogate this literature through the lens of neuroscience. Extending from a similarly new field of inter-disciplinary inquiry, neuroscience for architecture, we contend that this inquiry not only serves to reinforce (or question) the available evidence, but also identify targeted opportunities for future research.

## 2. Background

### 2.1. Trauma, Homelessness and Domestic Violence

Traumatic experiences are threatening, or potentially threatening, to an individual’s safety and survival. The World Mental Health Survey found that 70.4% of the world’s population have experienced a traumatic event in their lifetime. However, within the homeless population, the incidence of trauma is much higher [2]. Interviews of homeless persons in Sydney, Australia, revealed that 100% of women, and 90% of men had experienced trauma in their lifetime, with 58% having been physically assaulted, 55% witnessing serious injury or death, and 50% of women and 10% of men reporting having been raped [3]. Similarly, in the USA, approximately two-thirds of homeless women report having been beaten or raped by an intimate partner, and over 50% report that they have experienced sexual assault other than rape [4]. While the incidence of exposure to abuse and trauma is consistently higher for women [5], it is also a major factor leading to homelessness for men. For example, a survey of 25 homeless men in Ontario, Canada, found that all had experienced trauma in their childhood, and that this trauma contributed to their entry into homelessness [6]. 

Although only approximately 4% of the general population will develop trauma-related pathologies such as PTSD [1], there are substantially higher prevalence rates for people who have experienced homelessness and domestic violence. Whitbeck et al. [4] found that 42.6% of the 148 homeless women surveyed met the criteria for having had PTSD in their lifetime, and almost 30% had experienced this disorder in the previous twelve months. Similarly, Carlson et al. [7] estimated that 35% of homeless veterans would meet a diagnosis of PTSD, and in Australia, the 12-month prevalence of PTSD in a sample of 70 homeless people was 41%, and the lifetime incidence was 79% [8]. The experience of homelessness itself is also traumatic and can amplify the effects of prior trauma experiences [4,9]. Rape and intimate partner violence are also associated with a substantially increased risk of PTSD [2,10]. Unfortunately, the duration of post-traumatic symptoms—reliving the trauma, avoidance-behavior, hypervigilance, altered emotional responses, and relationship difficulties—can persist for many months and often years [2,11].

### 2.2. Trauma-Informed Design and Supported Housing

Trauma-informed design is founded on the more established field of trauma-informed care, relating and translating relevant key tenets to the design of the built environment. The first explicit set of principles were developed in 2018 by the Committee on Temporary Shelter targeting the design of environments for people who had experienced long-term homelessness [12]. Key criteria include [13] (p. 4):

- Reduce or remove known adverse stimuli;
- Reduce or remove environmental stressors;
- Engage the individual actively in a dynamic, multi-sensory environment;
- Provide ways for the individual to exhibit their self-reliance;
- Provide and promote connectedness to the natural world;
- Separate the individual from others who may be in distress;
- Reinforce the individual’s sense of personal identity; and
- Promote the opportunity for choice while balancing program needs and the safety/comfort of the majority.

Other expanded criteria and guidelines, such as those developed by the not-for-profit organization Design Resources for Homelessness [14], include an increased emphasis on safety, comfort, privacy and social connection.

Although still in its infancy, there is growing recognition of the importance of the design of the built environment as a key component of trauma-informed care [15,16]. The recent establishment of the Trauma-informed Design Society in the United States, founded by Roche, Cowart and Harte, seeks to translate research into practice and to assist organizations in creating ‘stress-reducing physical spaces’ [17]. The implications of a trauma-informed approach to design have been applied in a number of settings including female correctional facilities [15], residential treatment centers for children [16], and schools [18]. A recent publication by Schroeder et al. [19] has also introduced the notion of ‘trauma-informed neighborhoods’, aligned with research that contends that mental health and wellbeing is as much, if not more, closely linked with qualities of the broader neighborhood and community than with individual buildings [20].

Given the genesis of trauma-informed design in the homelessness context, there are also a number of studies that have explored the potential for trauma-informed design in relation to supported housing for people escaping homelessness [12,21,22]. Supported housing can be any type of housing that provides support for people with particular needs. While it is a loosely defined term, and overlaps with institutionalized contexts of supportive living, it emphasizes assistance for people to live as independently as possible within the community. McPherson et al. [23] developed a taxonomy of supported housing for mental health comprising five types across four domains encompassing level of support, location of support, physical setting (whether individual accommodation or congregate housing with communal facilities), and length of tenancy.

Within the context of homelessness and domestic violence, length of tenancy is the dominant classifier of supported housing types along a trajectory from emergency or crisis shelters and refuges (from one night to several weeks), to short-term or transitional housing (several weeks to several months) and ultimately permanent housing. However, building on the ‘Housing First’ model developed in the 1990s in the United States for people experiencing homelessness, there has been an increasing emphasis internationally on prioritizing direct access to permanent housing solutions. A key emphasis of the Housing First model is decoupling housing from support to offer greater choice and flexibility. Support can be targeted to resident need and may be offered more intensively on-site in congregate settings, such as in the ‘Common Ground’ housing model, or off-site. In the latter, tenancies are often dispersed in a ‘scattered site’ model and undifferentiated from other housing in the neighborhood in an effort to reduce visibility, stigma and concentrated disadvantage. In addition to supported housing, other accommodation solutions include temporary accommodation in hotels and motels, financial or other assistance to access private rental accommodation and pathways into social and public housing.

There are a number of studies that explore the design of the built environment in relation to homelessness. However, the literature targeting the design of supportive housing for women and children escaping domestic violence is relatively scant. An academic review by Grieder and Chanmugan [24] draws on the very limited available literature on the design of domestic violence shelters, together with therapeutic environment theory and the broader literature exploring evidence-based design in healthcare environments, to develop a set of design guidelines. Based on a model developed by Smith and Watkins [25], the guidelines are structured around four key design domains: sense of control, reducing or eliminating environmental stressors, enabling social support, and providing positive distracters. Interestingly, despite noting that safety was the ‘genesis’ of the domestic violence shelter movement, this aspect is largely absent in the guidelines, likely due to the adoption of a framework that has been developed from the healthcare context. However, in a recently published guideline targeting the design of refuges for women and children escaping violence in Australia, safety is foregrounded as the first of nine principles, with others encompassing the need for privacy, dignity, flexibility and accessibility, and an emphasis on children, sustainability, therapeutic space and sense of home [26]. The complex issue of safety in domestic violence shelters was also the catalyst for a project by the Washington State Coalition Against Domestic Violence in 2006, leading to a broader program exploring the design of domestic violence shelters and the establishment of Building Dignity in 2012, a design resource outlining aspirations, strategies and case studies for domestic violence housing [27].

### 2.3. Evidence-Based Design and Neuroscience for Architecture

While there is increasing recognition of the many ways that design of the built environment influences and affects health and wellbeing, questions remain about the robustness of the evidence. Drawing on the interdisciplinary field of environmental psychology, Evidence Based Design (EBD) attempts to address such concerns by ensuring that design decisions are informed by relevant and best-practice research [28]. However, research into the design of the built environment is constrained both by its position as a relatively new field of academic inquiry, and by the wide range of variables that cannot readily be ‘controlled’. Hence, studies are largely qualitative and cross-sectional and highly varied in terms of scope, methods, populations and contexts. As well as the limited and fragmentary nature of the available research, questions have also been raised about the robustness of methodology and the generalizability of findings, leading to calls for further research encompassing larger, representative samples, longitudinal studies and empirical verification of findings [13,29,30,31,32,33,34,35,36,37]. Such research would be welcome, but is constrained by the difficulty of representative studies in relation to the diversity of social, cultural, political and environmental contexts in which built environment design is enmeshed.

Neuroscience for architecture is a relatively new area of interdisciplinary inquiry (formalized with the establishment of the Academy of Neuroscience for Architecture in 2003). One key perceived benefit of neuroscience for architecture is to build a stronger empirical foundation in architecture and urban design, and to “move beyond speculating about what might be ‘better’ or ‘worse’ in terms of design” [38] (p. 479). To date, the junction of neuroscience and architecture has validated many of the disciplinary tenets of good urban design, such as the value of inhabitable edges and the importance of landmarks and patterns for orientation and navigation, as well as reinforcing the importance of direct and indirect connections with nature [39,40,41]. Further, this collaboration has provided insights into the relationships between the design of the built environment and health and wellbeing for specific populations with diverse needs and abilities; populations that are often not captured under a generic umbrella of ‘good’ design [42].

While we contend that such exploration can reinforce (or question) the available evidence, our primary intention is not to validate and legitimize prior knowledge. Nor is it to attempt to conflate two disciplines with vastly different knowledge bases and methodologies. Rather, we are interested in interrogating embedded narratives and assumptions from these respective fields and identifying targeted opportunities for future research that such insights might afford.

Thus, this research explores two key questions:

- What is the current evidence on the relationship between the design of the built environment and trauma in the context of supported and how does this correlate with established principles of trauma-informed design?
- What insights can neuroscience offer in extending or questioning our understanding of this evidence and what are the implications for future research?

## 3. Methods

Addressing the first research question, we undertook a review of the literature to identify the scope of existing evidence on the relationship between the design of the built environment and trauma in the context of supported housing. A scoping review process was adopted using the framework described by Arksey and O’Malley [43]. Scoping reviews provide an account of available research, but unlike systematic reviews do not attempt to synthesize literature in order to determine the relevant weight of evidence supporting particular approaches. They are more appropriate for studies with open-ended questions incorporating multiple study designs, and enable a greater degree of flexibility as the focus of inquiry is refined throughout the process [43].

Based on our existing knowledge of the field, we developed an initial set of search terms related to the three theme areas of trauma, design and housing. These were trialed in a series of pilot tests using the database Scopus. Relevant papers were mined to identify further common terms through reviewing titles, abstracts and keywords. Support from a University librarian was sought to refine and target the search terms and further pilot tests were undertaken to ensure an appropriate breadth of material was identified. Due to the fairly limited quantity of research, search terms related to trauma were expanded to incorporate ‘mental health’, and general, as well as typologically specific, terms related to housing were included. A full list of search terms is included in Table 1. These terms were used to conduct searches in the following databases: Scopus, Web of Science, CINAHL and Ovid Medline (encompassing PubMed and PsychInfo). Minor adaptations and customization of search terms were made to suit the requirements of particular databases. The search included all papers published up until and including January 2022.

Studies were initially selected by one researcher based on a review of titles. These were imported into bibliographic management software (Endnote X9.3.3) and duplicates deleted. Abstracts were then reviewed by one researcher against inclusion and exclusion criteria with a bias towards inclusion. Where inclusion was uncertain, abstracts were reviewed by the second researcher for confirmation. Inclusion criteria were as follows:

1. Directly related to the experience of trauma including both domestic violence and homelessness as well as trauma-related disorders such as PTSD;
2. Related to housing context including temporary, transitional and permanent housing;
3. Related to the design of the built environment;
4. Qualitative, quantitative or mixed methods studies with clearly identified methodology; and
5. Peer reviewed journal papers.

Exclusion criteria comprised:

1. Not explicitly related to experience of trauma including developmental disorders, psychiatric disorders, acquired brain injuries, depression and anxiety disorders, or the experience of staff rather than residents;
2. Institutional settings such as care homes, respite and places of incarceration, hotels and other travel accommodation, and any other non-residential setting such as workplaces, educational, recreational or other public buildings or spaces or healthcare environments;
3. Not directly related to the design of the built environment;
4. Literature reviews, position papers, or any research where research methods were not clearly stated, or papers that repeated/extracted findings from other included studies; and
5. Book chapters, conference papers, grey literature or non-peer reviewed papers.

Full papers selected for inclusion on the basis of abstract were then reviewed against the inclusion/exclusion checklist independently by both researchers and the final list of included papers determined by consensus. Reference lists of included papers were also screened for inclusion using a similar process.

**Figure 1:**
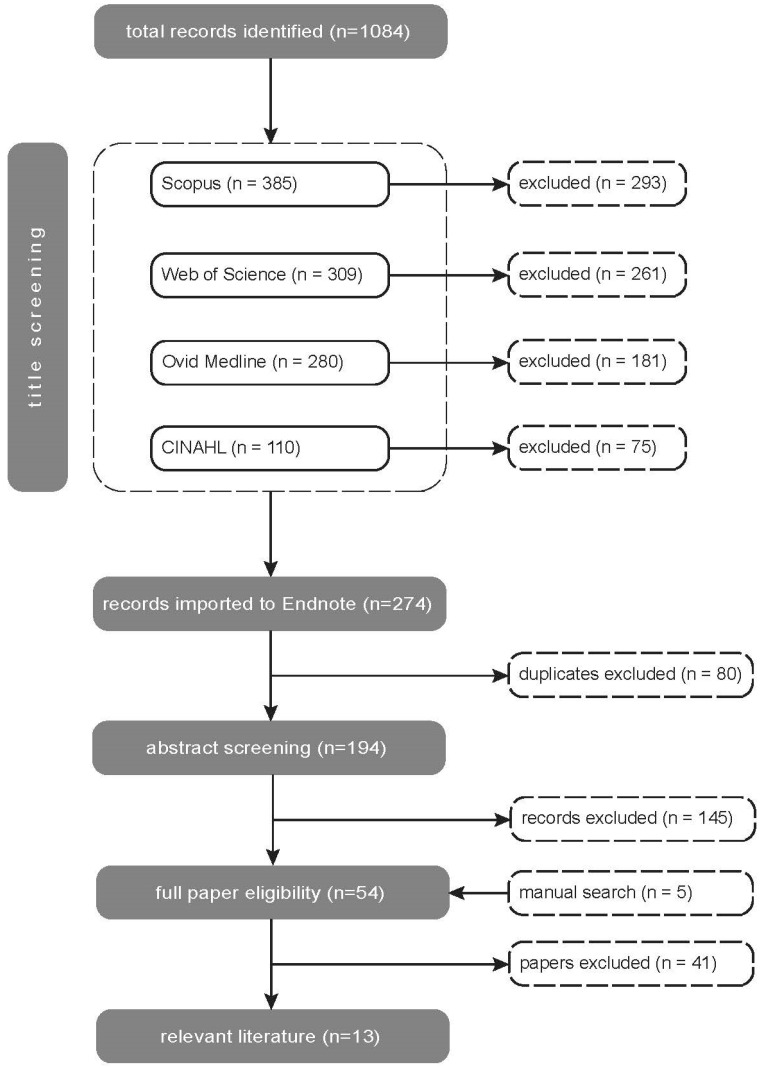
Diagram illustrating search and selection process.

A summary of the identification, screening and inclusion process is outlined in Figure 1. Of the total number of papers originally identified in the search [*n* = 1084], 810 were excluded based on review of titles, and a further 80 duplicates excluded. Of the remaining 194 papers, 145 were excluded based on a review of the abstract. The remaining 49 papers, together with a further five papers identified through reference lists, were then reviewed by both researchers against the inclusion/exclusion checklist. This resulted in a final list of 13 papers to be included in the review.

**Figure 2:**
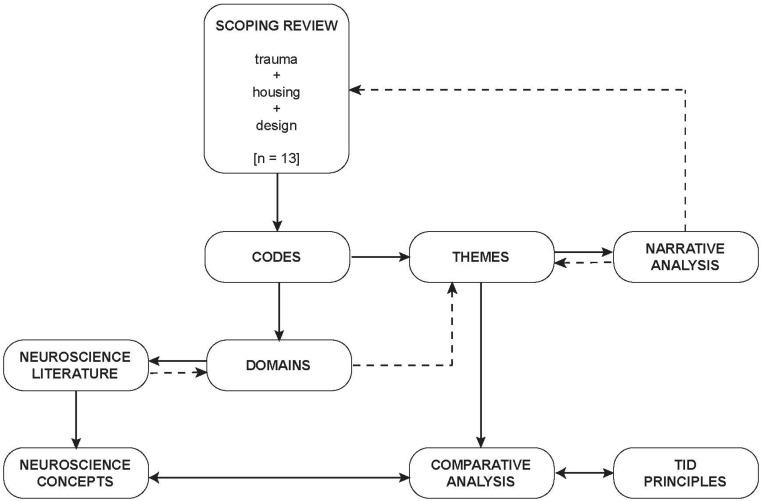
Diagram illustrating analysis process.

Since the focus of our paper is exploring the evidence-base for trauma-informed design, we chose to undertake an inductive process of coding rather than a framework analysis derived from trauma-informed design principles. The qualitative analysis followed a typical process as outlined by Lindgren et al. [44] emphasizing manifest content and a relatively low level of abstraction and interpretation. Each included paper was reviewed in detail by one researcher (architect) to extract key information related to the design of the built environment using descriptive codes. This information was charted using a Microsoft Word table to organize related codes. Through discussion and agreement by both researchers, these codes were organized under key domains that were also developed inductively. These domains were then related to neuroscience literature with brief summaries of relevant concepts prepared by one researcher (neuroscientist). Domains and codes that could not readily be interpreted in relation to neuroscience literature were excluded from further analysis and interpretation. Minor modifications to domains and refinement and organization of codes into themes under each domain was developed discursively by both researchers through multiple iterations until consensus was reached. A narrative analysis of the themes under each domain conducted by one researcher (architect) resulted in refinement of themes at a higher degree of abstraction and interpretation, but still deliberately targeting manifest content [45]. To confirm interpretation, this information was reviewed by the same researcher against the original text. As a final step, comparisons were made between neuroscience concepts and themes from the reviewed literature by both researchers through charting relationships in a Microsoft Word table. The relationship between the inductively developed themes and the principles of trauma-informed design was also assessed through comparison and interpretation. This was led by one researcher (architect) and confirmed by consensus with the second research (neuroscientist). An overview of the approach to the analysis is outlined in Figure 2.

## 4. Results

An overview of the 13 reviewed papers, including their key focus, study design, demographic focus and housing context, is outlined in Table 2. The papers ranged in publication date from 1989 to 2021, with the majority [*n* = 8] being published in the last five years. The overwhelming majority of papers are from the USA and Canada, with the remainder from Europe. Two sets of two papers are by the same authors, but were included as they related to different aspects of a larger study with different methods and/or contexts. The design of the built environment was the central focus for 8 of the studies, with the remaining 5 discussing the built environment as one aspect of a broader study focus. Study designs were predominantly qualitative with one mixed methods approach and four quantitative studies. Approximately half [*n* = 6] studies were based on single case study sites, with a further two comparing different housing contexts, four encompassing multiple housing locations, and one with no specific housing context. The housing contexts included permanent supportive housing [*n* = 3] and emergency and transitional housing [*n* = 9]. Demographics predominantly related to people escaping or at risk of homelessness [*n* = 9], followed by people escaping domestic violence [*n* = 3] and veterans diagnosed with PTSD [*n* = 1]. Demographics also encompassed parents, single and older adults, and gender and cultural diversity. No studies directly engaged children, but rather perspectives were based on the views of parents.

The initial coding and analysis identified three key domains relating built environment design to neuroscience comprising: Safety and Security; Control; and Environmental Enrichment. A fourth domain, Social Connection and Support, was excluded from further analysis due to the challenges of relating issues to neuroscience literature. These three domains are outlined below. Each section includes an introduction to the domain, a summary of key related concepts from neuroscience, a narrative overview of themes from the reviewed literature and a reflection linking the neuroscience concepts to the reviewed literature and trauma-informed design principles. A summary of the domains and related built environment design themes from the reviewed literature is outlined in Table 3. An expanded summary of the content of each reviewed paper in relation to the domains and themes is available in the Supplementary Information (see Table S1). A summary of the links between the domains and themes and trauma-informed design principles is outlined in Table 4. Strong links, where principles directly and comprehensively relate to themes, are indicated by dark grey and weaker links by light grey. Details of these links are discussed under each domain below.

### 4.1. Safety and Security

In simple terms, safety can be defined as situations and environments in which there is no or little chance of challenges to normal function of the body that could threaten survival. The ability to detect and respond to threatening stimuli or situations is essential for survival; however, equally important is the ability to learn that situations and environments are safe for exploration. Safety and security is also central to the experience of home, with Cooper Marcus [56] famously describing the house as a ‘mirror of self’ which acts as a “refuge from the outside world, a cocoon where we can feel nurtured and let down our guard” (p. 4). Thus, safety and security, is arguably the most fundamental aspect of trauma-informed design for housing.

#### 4.1.1. The Neuroscience of Safety and Security

There are highly developed neural circuits and processes dedicated to recognizing and responding to situations that could result in harm. Once a threat is detected, the brain initiates a complex response aimed at improving survival. The core of this response involves activation of the sympathetic (fight or flight) system and an increased release of glucocorticoids (cortisol in humans) from the adrenal gland that, together, prepare the body for any physical and mental challenges that might be encountered [57]. In addition, a collection of behavioral responses is observed; for example, passive-coping, characterized by a marked decrease in engagement with threat, and active-coping, characterized by persistent attempts to engage with (fight) or escape from (flight) the threat. When working appropriately, these three elements enhance an organism’s ability to survive life-threatening events [57,58].

Ideally, the activation of the ‘stress response’ is a direct result of exposure to a stressor; initiates change to successfully respond to the stressor; and stops when the stressor is removed, allowing the body to return to a resting state. However, the chronic activation of the stress response that results from prolonged exposure to threats is highly damaging to physical and mental health [57,58,59].

Although it is beneficial to respond to aversive events as they happen, it is arguably of more benefit to predict the occurrence of these events enabling individuals to prepare for, or preferably avoid, threats [60]. By learning to associate non-aversive ‘conditioned stimuli’ (e.g., lights, tone, odors, etc.) with the occurrence of aversive ‘unconditioned stimuli’ (e.g., discomfort, physical injury, etc.) the individual develops the ability to react to the conditioned stimuli, displaying a ‘conditioned response’ that might include passive (e.g., freezing) or active (e.g., avoidance) coping behaviors, increased sympathetic nervous system activation (e.g., increased heart rate), and changes in endocrine function (e.g., increased cortisol release) [60,61,62]. However, conditioned stimuli do not have to be discrete events such as tone or lights, they can also be the experience of the environment. In addition, fear memories can generalize over to features that are merely similar to the feature or features present when the aversive event took place [63]. This has the obvious advantage of allowing an experience in one context to initiate a conditioned response to prepare for potential threats in different but similar situations.

When functioning appropriately, the formation and expression of conditioned fear-memories helps us to deal with aversive events or avoid them altogether. However, dysfunction in the formation and expression of these memories underpins the development of maladaptive behaviors and disorders such as PTSD, where a conditioned response appropriate at the time of the trauma is no longer beneficial but actively detrimental to an individual’s well-being [63,64]. Psychological treatment for inappropriate responses to trauma-memories involves exposure therapy, where an individual is repeatedly and safely re-exposed to conditioned stimuli or memories of the conditioned stimuli (e.g., a specific object or location). The aim is for the individual to learn that they are now safe—that the stimuli or environments that once predicted threats no longer do so—leading to a suppression of the conditioned response seen upon recall of the fear-memory [65]. However, it is important to note that the fear-memory has not been erased. Instead, a new memory has been formed—that these stimuli no longer predict the occurrence of an aversive event. When this safety memory is activated, it suppresses the conditioned response previously seen upon recall of the fear-memory. Because of this, both exposure therapy and extinction are prone to spontaneous recovery (where the response to the fear-memory returns after some time), reinstatement (where the response to the fear-memory returns after exposure to the original, aversive ‘unconditioned’ stimulus), and reactivation (where the response to the fear-memory returns when the individual moves to a context different from where extinction training occurred) [66].

There are also other forms of safety learning [67]. A neutral stimulus (e.g., a tone) can become associated with the absence of an aversive event and so become a safety cue that signals that an aversive event will not happen. In addition, learned avoidance, where an individual learns to move to a safe location to avoid a predicted aversive event, is another form of learned safety. Learning that an environment/cue is safe or learning to avoid a predicted aversive event not only reduces the physiological and behavioral reactions seen during the recall of fear memories, it also produces positive emotional states that reward the attainment and occupation of safe spaces within an environment (e.g., a nest) [68,69]. Consistent with this, deficits in learning about safety and, as such, to make use of sources of safety in the environment is a key symptom of PTSD [69,70,71,72].

#### 4.1.2. Safety and Security and Design of the Built Environment in the Reviewed Literature

Of the thirteen reviewed studies, ten explicitly addressed relationships between safety and security and the design of the built environment. It should be noted that papers that did not directly address issues of safety and security had a targeted emphasis on health and wellbeing, including the role of gardens and the natural environment [52,53] and community spaces [21]. Two themes were identified encompassing defensible environments [*n* = 9] and environmental stressors [*n* = 8].

The construction of defensible environments relates to the ability to mitigate real and perceived threats to self and belongings. The literature revealed four specific design strategies to enhance the perception of control and feelings of safety and security comprising visibility, concealment, escape and secure boundaries.

The first strategy, visibility, fosters situational awareness and the anticipation of potential threats. Built environment design features include the ability to preview potential threats from outside through the provision of peepholes and buzzers at entries [49], and clear lines of sight through windows from interior to exterior [46,50,55]. Within shared housing environments, the importance of preview extends to the interior of the dwelling, including lines of sight into communal spaces [12,22]. In a study of veterans suffering from PTSD, Nuamah et al. [50] highlight aspects of interior layout and features that enhance situational awareness including open, circular layouts without ‘blind corners’ or the need to have ‘a back turned’ (p. 168) and spaces that are not ‘cluttered with furniture’ (p. 169). Visibility also encompasses opportunities for staff surveillance. While this conflicts with the following domain of control, Koen et al. [47] found that for some residents of a shared housing facility, particularly those who had experienced triggers for trauma within the building, social interaction with other residents would only occur in visible common areas or not at all. In a study of residents in a homeless shelter, McLeod and Walsh [49] also noted that privacy and the absence of staff surveillance was not universally preferred, with one participant even expressing a desire for supervision in the most private space of the bathroom to limit opportunities for perceived undesirable behavior by other residents.

Visibility contrasts with the second strategy of concealment—to see without being seen. Refuerzo and Verderber [55] highlight the importance of providing windows and views to the outside ‘without sacrificing occupant safety’ (p. 47). Lygum et al. [48] also discuss this complex relationship between visibility and concealment, where the openness of the garden space and the presence of lighting enhanced feelings of security for some, while for another resident, it was the ‘darkest corners’ that offered the most secure location to survey the environment while being hidden from view (p. 161). This notion of concealment is also extended to the building itself, through architecture that either blends into the surroundings [46] or buildings that are set-back and screened from public view [55]. Certain typologies may also disguise the presence of a shelter. However, as discovered by Datta [46] in the study of an emergency shelter located in a converted motel, this can create challenges with confused identities; in this case with the shelter being predisposed to unwanted and frequent visits from ‘single men in cars’ (p. 546).

While visibility targets awareness and anticipation of potential threats and concealment targets avoidance of threats, the final two strategies, escape and secure boundaries, emphasize protection from threats. Opportunities for escape include the provision of multiple and clearly identifiable paths of exit, larger transitional spaces, wider doors, and circulation spaces that are free of obstacles [12,50]. Secure boundaries include both the physical defensibility of the entire dwelling complex and the ability to protect personal space and belongings with solid walls and lockable doors and robust lockable storage [46,47,49,51].

The second theme targets the avoidance of the diverse range of visual, acoustic and olfactory environmental stressors that act as triggers for trauma. Sudden and unexpected noises are a common trigger, leading to recommendations for avoidance of alarms and good acoustic separation [47,50,55]. Other built environment design strategies include avoidance of fluorescent lights [12], the avoidance of odors that can act as triggers for trauma, and maintaining good air quality through enhanced ventilation [50]. Buildings and spaces themselves can become an environmental stressor. Bollo and Donofrio [12] describe how some people transitioning out of homelessness preferred to sleep outside in the courtyard garden of the supported housing development, while Nuamah et al. [50] outline the problem of the experience of claustrophobia for veterans due to the association of cramped spaces with combat. With private rooms typically being small in size, some studies emphasized strategies to enhance the ‘sense of space’ through the placement of windows and doors (including the previously mentioned ‘Dutch door’ example), the use of mirrors, and reducing visual clutter through storage and minimal furnishings [50,51].

Environmental stressors associated with the neighborhood can also impact feelings of safety and security, with one study highlighting the importance of positioning residences in quiet locations [55]. Two studies discussed the problems of locating residential facilities in areas that were seen to be ‘unsafe’ and ‘rundown’, but also noted the contrasting perceived advantages of these neighborhoods in access to services, supporting personal autonomy [46,49]. In McLeod and Walsh’s [49] study of shelters for older homeless women, residents also discussed the perceived value of non-judgmental communities in these neighborhoods. In one of the few quantitative studies in the scoping review, Refuerzo and Verderber [54] found that perceptions of neighborhood safety were strongly correlated with quality of sleep, the ability to relax and the perceived ability to attain personal goals. Feelings of security, for self and belongings, was also found to be strongly related to use and engagement in activities within the shelter environment [54].

#### 4.1.3. Safety and Security, Neuroscience and Trauma-Informed Design

As discussed above, threats can be broadly categorized as unconditioned (meaning that they could not be predicted) or conditioned (meaning that cues in the environment can help predict when the threat will occur). The features of conditioned fear-memories would seem to be particularly relevant to the design of built environments, in that re-experiencing a previously threatening environment, or key features of this environment, or features like those present when the fear-memory was formed, can lead to a suite of conditioned responses including increased physiological arousal, fight/flight behaviors, or disengagement [63]. However, it is easier to account for unconditioned threats because they are often experienced in a similar way by most people. Secure locks on doors and windows, security systems, reducing loud noise and extremes of temperature, reducing exposure to people who are distressed and potentially threatening, appropriate lighting, and providing opportunities to escape threatening or stressful situations are likely to convey a sense of safety to most people regardless of their background. However, reducing perceived threats and increasing a sense of safety when designing for those who have experienced trauma is more complex. Neuroscience has revealed that the memories formed during traumatic experiences often last indefinitely and extend beyond the key events related to the experience to incorporate sight, sound, smells, etc. that were simply present during the traumatic event. For designers, this means that predicting the features of a space that may trigger a traumatic memory can be next to impossible, keeping in mind that commonly the features linked to traumatic experiences may not be known even to the individual. As a consequence, the creation of defensible environments, with the multi-layered strategies of visibility, concealment, escape and secure boundaries, may remain critical to maintaining a sense of safety and security. Further, the built environment may also play a role in supporting and enhancing safety learning, through for example visible ‘safety cues’ and opportunities for retreat to a range of ‘safe spaces’, although this was not evident in the reviewed literature.

In relation to principles of trauma-informed design, as outlined in Table 4, there is strong alignment with the theme of environmental stressors and weaker alignment with the theme of defensible environments. Broadly, the first principle (reduce or remove adverse stimuli) relates to conditioned threats and the second (reduce or remove environmental stressors) to unconditioned threats. The sixth principle (separate the individual from others who may be in distress) relates to aspects of defensible environments, including the capacity to anticipate the threat through visibility and to escape the threat to a safe space with secure boundaries. However, connections to the strategy of concealment (of both self and building) and to more nuanced aspects of situational awareness are less evident.

### 4.2. Control

The perception of control can be interpreted as “the belief in one’s ability to exert control over situations or events in order to gain rewards and avoid punishments” [73] (p.2). Control and perceived control appear to depend on three pieces of learned knowledge: the probability that certain outcomes will occur, the ability to influence any outcome through action; and the extent to which desirable outcomes can be reliably achieved through action [74]. As Huys and Dayan point out “…by making rewards exploitable and punishments avoidable, control renders the world more pleasant, more colorful, and more worth exploring” (p.322). Thus, perception of control, with or without objective control, can rouse individuals to action, but a lack of perceived control can trigger or exacerbate many mental health conditions such as anxiety and depression [73,74,75]. A perceived lack of control is also identified as a key factor in the loss of a sense of home in institutional settings including residential care facilities [76], reception centers for asylum seekers [77] and transitional housing [78]. Given the connection between control and mental health, and in the context of transitional and supported housing, it is thus a key aspect of trauma-informed housing design.

#### 4.2.1. The Neuroscience of Control

First coined by Overmier and Seligman in 1967 [79], the term ‘learned helplessness’ was used to explain their finding that animals previously exposed to unavoidable shocks made no attempt to avoid the shock, even when they could (e.g., jumping to a safe location), but passively waited for the shock to end [79]. Crucially, humans exposed to inescapable, mildly aversive events (e.g., loud noise) also display learned helplessness, and learned helplessness soon became a leading explanation for the development of mental health conditions such as depression [80,81,82,83]. Indeed, not only did learned helplessness experiments produce symptoms of depression in both animals and humans, but people with major depression were also found to display learned helplessness (e.g., passivity) even if they had not received prior exposure to inescapable aversive events [82,83].

The assumption at the time was that both humans and animals expect to be able to exert some control over their environment, and when this expectation is violated they enter into a state of learned helplessness [84]. However, this idea has now been turned upside down with strong evidence that, by default, humans and animals expect to have no control over situations and environments [84]. Rather, when the ability to exert control is detected, neural circuits are turned on that suppress helplessness responses and promote attempts to actively engage with or avoid the situations/environments [84]. Hence, it appears that it is in fact the ability to control events, not the absence of control, that is detected by the brain, and the inability to detect options for control initiates the default helplessness response. Conversely, repeated exposure to the presence of control creates an expectation that events may be controllable in the future—a result that Maier and Seligman [84] suggests represents the ‘neural basis of hope’.

Humans (and animals) appear driven to exert control over their environment and the perception of control is beneficial for overall wellbeing, although illusions of control can also be detrimental (e.g., problem gambling) [85,86]. Indeed, Ly et al. [73] recently put forward a ‘reward-based framework of perceived control’, proposing that a perception of control is inherently rewarding and that this rewarding experience drives many of the improvements in wellbeing when individuals perceive control [73]. Further, they suggest that the reward associated with increased opportunities for choice acts to reinforce these choice behaviors and increase an individual’s perception of control. This proposal is particularly important in the context of trauma-informed design since it has been repeatedly shown that being able to exert control in one context can influence an individual’s perception of control in other, unrelated contexts [73,87,88].

#### 4.2.2. Control and Design of the Built Environment in the Reviewed Literature

Control in relation to the design of the built environment was discussed in ten of the thirteen studies. Two themes were identified, constituting self-reliance [*n* = 7] and territory [*n* = 9].

Self-reliance encompasses aspects of the built environment that are seen to support (or hinder) agency and independence in conducting everyday activities and interactions. Independence can be fostered or constrained by the location of the housing in relation to proximity to services, amenities and access to transport [55]. For parents with young children, housing design that enables passive surveillance affords opportunities for greater self-reliance through the ability to get things done [46,55]. Examples of self-reliance in everyday activities ranged from the simple act of controlling light switches [12], to growing produce for consumption and access to kitchens and equipment [21].

The value of independence in undertaking everyday activities is more than transactional. For example, in Huffman’s study [21], the garden not only offered benefits of access to ‘free’ food, but also educational opportunities and a sense of self-worth through hard work and ‘getting dirty’. In contrast, the importance of personal hygiene and the ability to ‘keep clean’ was highlighted as an important aspect of dignity, particularly for people escaping homelessness [22]. As well as access to bathroom facilities, several studies emphasized the importance of organization and cleanliness of the built environment itself [22,48,55], including the importance of storage in enhancing perceptions of control [46,51].

The perception of control is a key factor in the second theme, the control over territory. Several studies identified the importance of designated personal space, particularly for the most private of activities in bathing and sleeping, with the provision of separate, lockable rooms [47,49,55]. However, more nuanced forms of control are enacted through permeable and flexible boundaries. For example, Pable [51] describes how a lockable ‘Dutch door’ and the installation of curtains around beds substantially enhanced perceptions of privacy in a shared family bedroom, while enabling control in levels of connectivity with family members, staff and other residents. Within the shared bedroom, the control over personal space is also enhanced through opportunities to display personal items, which act as markers of claims to personal territory [51].

Control in communal spaces is particularly complex as these can become sites of conflict [21]. Recommendations to minimize conflict include the provision of larger areas and multiple distinct spaces [12] or zoning and screening within single larger spaces [22]. However, as McLane and Pable’s [22] detailed study of the design and use of community spaces in supportive housing revealed, these are typically underutilized by residents.

One key factor limiting use are institutional policies, including imposed rules of the institution [47] as well as uncertainty over individual rights of access [48]. The limits of individual freedom within the confines of the institution is a tricky negotiation, where safety, security and the needs of many (including staff and other residents), must also be considered. However, perceptions of the extent of control are directly related to the design of the built environment and are enhanced through visibility and connectivity of spaces [22], flexibility in space and furniture inviting different uses [12,55], and the design of décor and elements that enhance privacy in public spaces [48] and mitigate the experience of spaces that are ‘overpoweringly institutional’ [22] (p. 47). For example, Bollo and Donofrio [12] describe how open and accessible reception desks foster a sense of ‘trustworthiness’. Gardens and external spaces also offer opportunities for enhanced perceptions of control. As Lygum et al. [48] (p. 161) describe, these are seen as ‘free spaces’, particularly for children.

#### 4.2.3. Control, Neuroscience and Trauma-Informed Design

Two aspects of the neuroscience of control that can inform trauma-informed design are the discovery of the ‘neurocircuitry of hope’ and the related concept of a reward-based framework of control and perceived control. Together, these speak to the importance of providing and allowing opportunities for residents to exercise (or at least have the option to exercise) control over their space and social interactions. According to the reward-based framework of control, activating reward circuitry within the brain through regular opportunities to exercise control positively reinforces these behaviors, driving individuals to pursue further opportunities to exert control. This, in turn, might strengthen the neural circuits for detection of control that are responsible for stimulating active responses to challenges while simultaneously inhibiting passive responses that lead to learned helplessness. The evidence points to some important elements that act as signals of perceived control including the presence of storage, flexible furniture and operable barriers (curtains, doors). The evidence also posits that the design of objects and spaces can promote a sense of ‘trustworthiness’ (e.g., reception desks) and ‘freedom’ (e.g., the garden), although these remain less clearly defined.

In relation to principles of trauma-informed design, as outlined in Table 4, there is strong alignment and direct connection between the theme of self-reliance and the fourth principle (provide ways for the individual to exhibit their self-reliance). Control over territory (both personal and common space) is not explicitly referenced, but is related to both the seventh principle (reinforce the individual’s sense of personal identity) and the eighth principle (promote the opportunity for choice).

### 4.3. Enriched Environments

The last domain, enriched environments, emphasizes the importance of providing individuals with opportunities to engage in complex environments composed of diverse sensory, motor, cognitive, and social experiences. Unlike the previous domains, this concept is derived directly from neuroscience. Within animal models, this represents a departure from the sparse housing usually found in laboratories to include access to toys, nesting materials, running wheels, and a larger home environment with multiple, non-threatening cage-mates [89,90]. While the exact make-up of each enriched environment varies, almost all studies report beneficial effects [91,92]. ‘Enrichment’ is not a term that is used explicitly in relation to housing, but arguably permeates all aspects as the foundation of ‘good’ design.

#### 4.3.1. The Neuroscience of Enriched Environments

Environmental enrichment alters neuronal and non-neuronal cell function, enhances neuronal plasticity and neurogenesis, alters production and release of neurotransmitters (e.g., serotonin, dopamine, noradrenaline), produces improvements in learning and memory, improves regulation of stress responses, and has anxiolytic and anti-depressant effects [90,92]. However, the changes seen in animal models may reflect not only exposure to enriched environments but also a reduction in the negative effects of the poorly enriched housing of the usual laboratory animal [91,93].

According to the ‘inoculation stress hypothesis’ [94], the stress-reducing and anxiolytic/antidepressant effects of environmental enrichment result from the constant exposure to novel and mildly stressful experiences that these environments provide. Regular exposure to these low-level stressors produces a collection of neural and non-neuronal adaptations that enable individuals to cope more effectively with current and future stressors [94,95]. In addition, enriched spaces provide opportunities for individuals to exert control over their environment, with a recent review by Rojas-Carvajal et al. proposing that “the fact that rats can manipulate objects and materials at different configurations induces exploration and increases the ability to control and predict what is happening in the surroundings, which in turn reduces anxiety and improves animals’ welfare” [91] (p. 17). In addition, enriched environments appear to be intrinsically positive and rewarding, with rats displaying a preference for environments with novel objects, even learning to press a lever to gain access to running wheels or opportunities for social interaction [96,97]. Extending this research beyond the laboratory setting, Rojas-Carvajal et al. [91] propose that animal laboratory housing models can be adapted to explore the stressors of our urban environment, emphasizing the stress-relieving benefits of connection with nature.

#### 4.3.2. Enriched Environments and Design of the Built Environment in the Reviewed Literature

Enriched environments was the most prevalent domain within the reviewed literature, discussed in twelve of the thirteen included studies. Three themes were identified comprising connection to nature [*n* = 8], environmental diversity [*n* = 5] and image [*n* = 6].

Under the first theme, the natural environment was the exclusive focus of three papers in this scoping review [48,52,53], underscoring the role of nature as a therapeutic setting and the benefits of direct interaction with flora and fauna. In their quantitative study of parents living in shelters, Peters et al. demonstrated that direct connections with nature significantly affected psychological need fulfilment [53] and was associated with higher parental need satisfaction and lower parental need frustration, particularly for parents with young children [52]. Huffman [21], discussing the role of community spaces in supporting wellbeing in permanent supportive housing, describes how the food garden offers both peace and quiet, as well as positive childhood memories for some residents. Several other papers outlined the importance of integrating gardens and ‘outdoor rooms’ within residential developments, to support connections with natural environments [12,48,52,53,55]. Beyond direct connections to the outdoors, papers highlighted the benefits of indirect connections with nature including daylight [12,22,50,55], openable windows and access to fresh air [50], and views of trees and planting as calming environments [12,22,50,55]. While not strongly evident in the literature, there was some reference to more abstract connections with nature, with one participant in Nuamah et al. [50] study of veterans with PTSD noting a preference for the color green, which “makes me feel calm” (p. 169).

In contrast to the stated preference for the color green by one of the participants in Nuamah et al.’s [50] study of veterans with PTSD, more than 40 percent articulated a preference for ‘brighter colors’ over ‘monochromatic bland color palettes’ [50] (p. 169). This relates to the second theme, environmental diversity, encompassing intensity and multiplicity of sensory experience. Natural environments also offer benefits in this regard. In Lygum et al.’s [48] study of a crisis shelter garden, the opportunities of immersive sensory experiences, and the desire for “more sources of sensory qualities, such as fruit, colors and smells”, were identified by staff and residents (p. 161). The garden was also seen as a site of opportunity to respond to diverse needs and responses of children who have been exposed to trauma, through enabling ‘many ways to play’ [48]. In internal spaces, diversity can be accommodated through the design of discrete spaces targeting different sensory experiences, for example through the provision of multiple common rooms [12]. The affordance of individual elements can also invite diversity in occupation, activity and interpretation, particularly for children, acting as ‘vehicles for imagination’ [51] (p. 23).

The importance of diversity is underscored by differences in personal preferences, interests and needs. Thus, arguably the pinnacle of diversity is personalization. Opportunities for personalization discussed in the literature include moveable furniture, artwork, photographs, shelving and pinboards for the display of personal items, [12,46,51]. While typically these built environment features are discussed under the theme of control, they also present opportunities for the creation of rich environments, as occupied territory can be uniquely targeted to the needs and tastes of the individual occupant. In Pable’s [51] study of an altered shelter bedroom pre- and post-occupancy, a significant increase in personalization was observed following the introduction of ‘prompts’ designed to invite personalization.

The last theme, ‘image’ relates to the overall appearance of the housing, and its alignment with individual and societal expectations. A number of papers emphasized the importance of the creation of ‘homelike’ environments [22,49,55]. While used somewhat loosely, together with terms such as ‘hominess’ and ‘cheery’, they are used to convey an aesthetic of warmth, comfort, and ‘richness’, posited as an antithesis to more clinical and impoverished institutional environments. This aesthetic of home is underpinned by two key strategies—the avoidance of institutional features such as large buildings and parking lots [55] and the presence of ‘homelike’ features through décor, soft furnishings, and objects such as flowers and books and pets as well as qualities such as appropriate lighting and cleanliness [22,49].

The aesthetic of ‘home’ was found to be a key factor in the use of common spaces [22]. More broadly, correlations between use and the appearance of the built environment, including interior, exterior, and neighboring buildings, were identified by Refuerzo and Verderber [54] in their larger quantitative study of underlying determinants of residential satisfaction in shelters for victims/survivors of domestic violence. The appearance of the shelter and the neighboring environment, were also shown to affect feelings of personal status, and both status and use of the shelter were associated more broadly with quality. While perceptions of quality extend beyond appearance, in Refuerzo and Verderber’s study, again, this was directly connected to the presence of certain features such as windows, views, quality furnishings, and trees and vegetation [54], as well general cleanliness and maintenance of the shelter [55]. The relationship between appearance and quality was also revealed in Huffman’s [21] study of a purpose-built permanent supportive housing project for people escaping homelessness. Countering the dominant concept of ‘hominess’, two participants articulated the value of the large glazed open communal areas as transporting you to somewhere ‘else’, specifically for one participant the Grand Canyon, and the other a ‘hotel in New York.’ However, perceptions of home and quality are inherently situated and relative, resulting in contrasting experiences of living in a shelter as a move up or down in the world [46].

The theme of enriched environments thus integrates a complex array of characteristics that encompass opportunities for encounters with a multiplicity of sensory experiences, environments that can be uniquely targeted to individual needs and preferences, and environments that elicit positive associations, whether in relation to individual memories, societal norms and aspirations, or innate preferences for connection with nature.

#### 4.3.3. Enriched Environments, Neuroscience and Trauma-Informed Design

Environmental enrichment is a term that is largely confined to the neuroscience literature, although it is beginning to make headway in psychology and other areas of mental health (e.g., dementia). While a sizeable body of neuroscience research exists highlighting the benefits of positive social interactions and environments that are more stimulating (albeit minimally) than a bare space, neuroscience has far less to say on the benefits of engagement with nature, and the impact of different colors, smells and textures on brain function and mental health. In contrast, architecture and design literature, as well as a wealth of multi-disciplinary research from geography and planning, sociology, public health, nursing, and psychology, has demonstrated the multiple health benefits of connection with nature, including reduced stress, psychological distress and depression, enhanced memory and cognitive development, and the promotion of general wellbeing [98,99,100,101,102,103,104,105,106]. Conversely, a lack of green space, has been shown to negatively impact mental health and wellbeing [107].

Not surprisingly, connection to nature is clearly identified as a central principle in trauma-informed design. Similarly, as outlined in Table 4, the second theme, ‘environmental diversity’, also relates directly to the third principle, ‘engage the individual actively in a dynamic, multi-sensory environment’, and has connections to two other principles, highlighting its importance to and prevalence within design. The third theme, ‘image’ is not explicitly referenced, but relates to the first principle ‘reduce or remove adverse stimuli’ in terms of the avoidance of institutional features (that may be a trigger for trauma) and to the last two principles through reinforcing alignment of the design of the built environment with personal identity and promoting opportunities for choice to meet the diversity of experiences and expectations.

## 5. Discussion

In this paper we have explored the evidence for trauma-informed design of supported housing through the lens of neuroscience. We sought to establish the extent of the current evidence through a targeted scoping review, and to relate this evidence to trauma-informed design principles and to key themes from the neuroscience literature to determine what insights it can offer and the implications for future research.

The findings from the scoping review establish that the existing literature is limited in scope, largely qualitative in nature and very limited in geographic context. Since we were only able to include studies published in English, we recognize that this may be a limitation of this scoping review. However, interest in this field of research is increasing, with the majority of studies published in the last five years and a large number [*n* = 6] published in the last two years. A particular gap of note is understanding the experiences and needs of children, with no studies that met the inclusion criteria for this scoping review, and very little literature more generally, directly engaging children and young people who have experienced trauma in research about their experience of the built environment. Further, as Pable [51] contends, both young children and adolescents “May be particularly impacted psychologically by physical environmental conditions” (p. 34).

However, the commonality of themes across studies lends support for the generalizability of findings. As shown in Table 4, there is also substantial alignment between the domains and related themes and the principles of trauma-informed design. In particular, our inductively developed themes of environmental stressors, self-reliance, connection to nature and environmental diversity are clearly and explicitly related to the established principles of trauma-informed design. The gaps noted above, including defensible environments, control over territory, and image, are more evident in the expanded ‘human needs’ principles and design goals outlined in the guidelines developed by Design Resources for Homelessness [14]. Additionally, this resource encompasses connections to our fourth (excluded) domain Social Connection and Support. These additions are evidence of the maturing of a nascent field of design practice. However, we contend that there are further opportunities to expand the principles and design criteria, particularly in relation to the domains of Safety and Security and Control. Additionally, this will be an ongoing and long-term project as (hopefully) over time the evidence-base for trauma-informed design expands.

Although the domains of Safety and Security, Control, and Enriched Environments are useful as an organizing structure, there are many overlaps and connections between the domains, themes, and specific features of the built environment. For example, cleanliness relates to a sense of control (e.g., being able to manage personal hygiene through access to appropriate facilities and the ability to store and organize belongings), but also to safety and security (e.g., reducing visual clutter and enhancing situational awareness) and to enriched environments (e.g., the creation of a positive image and perceptions of quality). Aspects of control are similarly fundamental to safety and security, for example in the provision of dedicated personal space that enables the construction of secure boundaries. Further, control over territory, enacted through the display of personal items and organization of furniture, contributes to environmental enrichment through the creation of diverse personalized spaces uniquely targeted to the needs and preferences of individuals.

The overlap and alignment between domains and themes can result in single built environment design elements offering multiple benefits. For example, storage affords opportunities for maintaining control and order and for marking territory in personal space, safety and security for belongings, and environmental enrichment through the display of personal items. However, other aspects of built environment design are more complex; for example, managing potential conflict between the creation of diverse, enriched environments in common spaces and in enhancing perception of safety and security through establishment of defensible spaces and reducing sensory load. As discussed under the domain of safety and security above, opportunities for visibility and concealment must be carefully managed in the design of the built environment, and as noted by Lygum et al. [48] in their review of a shelter garden space, the provision of safety through secure boundaries can also lead to feelings of confinement. Conflicts between control and security are also noted by McLane and Pable [22], with the deliberate limitations placed on access to common spaces for the benefit of resident safety described as an ‘odd predicament’ (p.43).

As noted above, similar issues and conflicts are identified in the broader literature, including navigating the complexities of expectations of freedom and control of the domestic space within the confines of the institution. In relation to safety and security, prospect-refuge theory [108] posits that our built environment preferences for spaces that offer both opportunities for visibility (prospect) and concealment (refuge) are biologically constituted. If such preferences are universal, if a sense of control, safety and security are part of the axiomatic understanding of home, and ‘enriched environments’ a foundation of ‘good’ design, how are they differentiated in the context of trauma?

Extending and questioning our understanding of this evidence through the lens of neuroscience has highlighted some potential gaps and opportunities for future research.

First, in relation to safety and security, is the distinction between conditioned and unconditioned stimuli and the implications for trauma-informed design. Designing for conditioned stimuli is complex due to both difficulties in predicting features that may trigger a traumatic memory, and the endurance and transferability of fear memories. While understanding what is known and being discovered about how specific stimuli and environments become associated with trauma and trigger fear responses will be valuable in ‘reducing or removing’ adverse stimuli and environmental stressors, arguably, even greater emphasis needs to be placed on creating defensible environments, not only in crisis shelters and accommodation, but also within permanent supported housing. Golembiewski [42], in a study exploring the neuroscientific basis of mental illness in relation to the design of the built environment, similarly identifies safety as the most essential aspect of environmental design. While certain common built environment features were identified, such as those promoting situational awareness, control over social interactions, pathways for escape, and avoidance of particular environmental triggers such as noise, they also contend that environments need to promote a ‘compelling and positive story’ that suggests safety and comfort [109] (p. 10). This is a complex area that likely encompasses a synthesis of an array of built environment qualities and substantial variation between individuals depending on the context of the trauma experience. Although some aspects of the built environment that act as adverse conditioned stimuli may readily be identified and eliminated, others may not. Alternatively, despite the refractor nature of fear-memories, the integration of safety cues may modulate the effects of adverse conditioned stimuli through repeated exposure over time. Future longitudinal studies could explore the connection between the design of the built environment and feelings of safety and security in relation to specific trauma contexts, conditioned safety cues, and the transferability and efficacy of these cues over time.

Similarly, the reward-based framework of control and perceived control and the ‘neurocircuitry of hope’ highlights the importance of providing multiple and diverse opportunities for exerting control for people who have experienced trauma. Therefore, while increasing opportunities for residents to exercise control over their environments can present organizational, financial, and safety challenges, we contend that designers place opportunities for control at the forefront of their thinking. These opportunities, however small, can create environments where people want to stay and help to combat feelings of helplessness that can result from exposure to trauma. Although small in scope, Pable’s (2012) study of the relationship between internal control and the design of the built environment through targeted alterations to a homeless shelter bedroom suggests that particular features in the built environment can extend a sense of internal control for people who have experienced trauma. Future research might extend understanding of the design of spaces and elements that enhance the perception of control. For example, are there differences between simple and clear acts of control (e.g., switching on a light, opening or closing of a Dutch door) and more nuanced forms of control (such as reorganizing furniture and the display of personal items)? How do these vary across individuals, populations (e.g., young children, adolescents and adults), and across the trajectory of recovery from trauma? How are perceptions of control transferred across environments, and what are the implications for design for both emergency accommodation and for permanent housing? And how might specific acts of control be extended to communicate more abstract associations such as ‘trustworthiness’ and ‘freedom’?

The third domain highlights the importance of environmental design within a salutogenic rather than pathogenic framework. As noted above, the overlap between environmental enrichment and control offers inherent benefits in the creation of environments that are uniquely suited to an individual’s needs. However, another key aspect of environmental enrichment is the value of novelty and exposure to mildly stressful experiences that not only enhance cognitive functioning, but also increase capacity to manage current and future stressors. Connection with nature is a central theme in environmental enrichment, and in the broader literature on health and wellbeing. The dominant narrative positions connection with nature as restorative and stress-relieving; however, it may be equally valuable as a positive stressor (or eustressor). These are not necessarily conflicting, and the ‘enriching’ qualities of natural environments are also discussed in the literature, building on Kaplan’s conceptualization of ‘soft fascinations’ [110] (p. 172). However, future research might specifically investigate this relationship between restoration and stimulation and the implications for the design of the built environment, including the various forms connection with nature can take, from immersion, to views, daylight or abstract associations with nature through natural materials, colors, forms and patterns.

While we contend that neuroscience can serve to reinforce evidence and further interrogate assumptions in built environment design research, we also recognize the limitations. Neuroscience is necessarily narrower in focus and cannot encompass the diversity of human-environment interactions. As such, our discussion of the evidence for trauma-informed design is inevitably partial, including our exclusion of the domain Social Connection and Support from our analysis.

## 6. Conclusions

Although limited in scope, the existing literature on trauma, design and housing is relatively consistent and aligned with the principles of trauma-informed design. However, insights from neuroscience offer some interesting opportunities for future research to target investigations in questioning and extending our understanding of the relationship between the design of the built environment and trauma. Our study has identified three potential areas of future research including the relationship between specific trauma contexts and conditioned safety cues in the built environment and the transferability and efficacy of these cues over time; how specific built environment cues might activate neural circuits for recognizing control (the ‘neurocircuitry of hope’), and the transferability of these cues across time and contexts; and the opportunities of connection with nature in trauma-informed design through explorations of restoration and stimulation in relation to the inoculation stress hypothesis. Additionally, our research has highlighted potential implications for trauma-informed design practice, including the centrality of defensible environments, and the importance of embedding multiple and diverse opportunities for exerting control in the design of the built environment.

Formalizing the intersection of built environment design with neuroscience (e.g., establishment of The Academy of Neuroscience for Architecture) has provided considerable insights into both established and emerging design principles. We hope that highlighting this relationship in relation to design and trauma will stimulate further collaboration between architects, designers, and trauma-focused neuroscientists and psychologists. We believe that these collaborations can stimulate the development of improvements in supported accommodation for people with a history of trauma. However, we also contend that benefits would flow in both directions, helping neuroscientists develop a better understanding of the complexities of trauma care and support in real-word settings, an understanding that could lead to improvements in models of trauma and investigations of novel treatment options.

## Figures and Tables

**Table 1:** Search Terms.

Theme Area	Search Terms
Trauma	“trauma-informed care” OR “trauma-informed practices” OR “trauma-informed design” OR “post-traumatic stress” OR “PTSD” OR “domestic violence” OR “partner violence” or “family violence” OR “interpersonal trauma” OR “traumatic violence” OR “child abuse” OR “childhood adversity” OR homeless * OR “mental health”
Design	“architecture and design” OR “architectural design” OR “physical design” OR “spatial design” OR “space design” OR “built environment design” OR “housing design” or “shelter design” or “refuge design” OR “interior design” OR “landscape design” OR “design features” OR “design elements” OR “built environment features” OR “built environment properties” OR “built environment characteristics” OR “environmental characteristics” OR “physical environment” OR “housing environment” OR “place attributes” OR “person-environment relationships” OR “trauma-informed design”
Housing	“supportive housing” OR “permanent housing” OR “temporary housing” OR “transitional housing” OR “crisis shelter” or “emergency shelter” OR “temporary shelter” OR “transitional shelter” OR shelter OR refuge OR hous* OR home OR residen*

**Table 2:** Overview of the reviewed literature.

Reference	Country	Key Focus	Study Design	Demographic	Housing Context
Bollo & Donofrio, 2021 [12]	USA	Application of Trauma-informed Design (TID) principles in the common areas of permanent supportive housing.	Qualitative case study: walking interviews with staff, environmental observations, and analysis of floor plans and meeting minutes of trauma-informed design renovation committee. No interviews with residents, but ‘evidence of use’ analysed through environmental observations and meeting minutes included resident representation on committee.	Formerly homeless people	Comparison of four ‘primary’ (TID) case study buildings and four ‘negative’ case study buildings. Primary study buildings comprise recently renovated [*n* = 3] and purpose-built [*n* = 1] permanent supportive housing (ranging from 46-84 units) with on-site support services.
Datta, 2005 [46]	USA	The relationship between feelings of ‘home’ and ‘homelessness’ and the architecture of emergency homeless shelters.	Qualitative case study: in-depth interviews with residents [*n* = 10] and environmental observations of common spaces (external, offices, childcare).	Homeless families	Single study site: emergency shelter in converted ‘run-down’ motel (30 families) with on-site support services.
Huffman, 2018 [21]	USA	Role of community space in supporting wellbeing in permanent supportive housing.	Qualitative case study: immersion in context (participation and engagement in meetings and activities) and in-depth interviews [*n* = 26]	Formerly homeless	Single study site: purpose-built permanent supportive housing (100 residents) with on-site support services
Koehn et al. 2020 [47]	Canada	The impact of trauma histories on the experiences of daily living and health outcomes for people living with HIV in supportivehousing environments.	Qualitative study: semi-structured interviews (24 participants)	Adults living with HIV who have experienced or are at-risk of homelessness.	Single study site: purpose-built permanent supportive housing (large facility) with on-site support services
Lygum et al., 2019 [48]	Denmark	Testing of guidelines for gardens to support health and wellbeing in crisis shelters for women and children exposed to domestic violence.	Qualitative case study: post-occupancy evaluation including analysis of landscape design, environment observation (physical traces), walking semi-structured interviews with staff [*n* = 12] and residents [*n* = 3] and participatory design/education program with staff.	Victims/ survivors of domestic violence	Single study site: crisis shelter in newly renovated historic building including evidence-based garden design (17 families) with on-site support services.
McLane & Pable, 2020 [22]	UK and USA	The design of interior communal spaces in supportive housing to aid in recovery from trauma.	Mixed methods case studies: exploratory questionnaires [*n* = 28], open-ended interviews [*n* = 18] with both residents and staff, space syntax analysis, and environmental observation via photography.	People escaping homelessness or at-risk of homelessness	Two study sites: transitional supportive housing in modified existing buildings with mix of shared and private rooms.
McLeod & Walsh, 2014 [49]	Canada	The experiences of women who become homeless after age 50 and the implications for shelter site, situation and service delivery.	Qualitative study: in-depth interviews [*n* = 8].	Older homeless women	Multiple temporary shelters with on-site support services.
Nuamah et al., 2021 [50]	USA	Identifying architectural and space design considerations and guidelines for veterans diagnosed with PTSD.	Qualitative study: in-depth semi-structured interviews [*n* = 17].	Veterans diagnosed with PTSD	No specific site: participant reflections on ‘general buildings’ including ‘both public and private spaces’ and ‘ideal living area’.
Pable, 2012 [51]	USA	Relationship between the design of homeless shelter bedrooms and perceptions of internal control, crowding and privacy.	Qualitative case study: comparison of design intervention in private dormitory bedroom (pre and post occupation) [*n* = 1] and continuous occupation of an unaltered private dormitory bedroom [*n* = 1] employing self-directed photography and in-depth interviews with occupants [*n* = 2], quantitative self-report questionnaire measurement [*n* = 2], photographic observation by researcher, and interviews with case-study managers [*n* = 2].	Single mothers with children escaping homelessness	Single study site: transitional homeless shelter (20 bedrooms).
Peters et al. 2020 [52]	Netherlands	Impact of natural environment on wellbeing of parents in homeless shelters.	Quantitative study: comparative questionnaire of parental need satisfaction and need frustration and connectedness with nature during personalized ‘nature experience’ intervention and in ‘standard indoor environment’ [*n* = 160] and staff questionnaire outlining activity and observations.	Parents of children escaping homelessness	Multiple study sites: women’s and/or homeless shelters.
Peters et al., 2021 [53]	Netherlands	Impact of personalized exposure to natural environments on wellbeing of parents in homeless shelters.	Quantitative single case experiment: repeated and randomized exposure to indoor environment (baseline phases) and natural environment (intervention phases) for parents residing in shelters, self-report questionnaire on psychological need fulfilment and wellbeing, and affective state (alter report) by researcher. [*n* = 3]	Parents of children escaping homelessness	Single site: transitional homeless shelter for families.
Refuerzo & Verderber, 1989 [54]	USA	Identifying underlying determinants of satisfaction for residents and staff in shelters for victims of domestic violence.	Quantitative study: survey with residents [*n* = 51] and staff [*n* = 50].	Victims/ survivors of domestic violence	Multiple study sites: temporary shelters for women and children [*n* = 6].
Refuerzo & Verderber, 1990 [55]	USA	Perceptions of environment for residents and staff in domestic violence shelters.	Quantitative study: photo-questionnaire/survey with residents [*n* = 51] and staff [*n* = 50].	Victims/ survivors of domestic violence	Multiple study sites: temporary shelters for women and children [*n* = 6].

**Table 3:** Summary of Domains and Themes in Relation to Reviewed Literature.

		Bollo & Donofrio, 2021 [12]	Datta, 2005 [46]	Huffman, 2018 [21]	Koehn et al. 2020 [47]	Lygum et al., 2019 [48]	McLane & Pable, 2020 [22]	McLeod & Walsh, 2014 [49]	Nuamah et al., 2021 [50]	Pable, 2012 [51]	Peters et al. 2020 [52]	Peters et al. 2021 [53]	Refuerzo & Verderber, 1989 [54]	Refuerzo & Verderber, 1990 [55]
**Safety and Security**														
Defensible environments		•	•		•	•	•	•	•	•				•
	Visibility	•	•		•	•	•	•	•					•
	Concealment		•			•								•
	Escape	•							•					
	Secure boundaries		•		•	•		•		•				
Environmental stressors		•	•		•			•	•	•			•	•
	Residence	•			•				•	•				•
	Neighbourhood		•					•					•	•
**Control**														
Self-reliance		•	•	•		•	•			•				•
Territory		•		•	•	•	•	•		•			•	•
	Personal space		•		•			•		•			•	•
	Common space	•		•	•	•	•							•
**Enriched Environments**														
Connection to nature		•		•		•	•		•		•	•		•
Environmental diversity		•	•			•			•	•				
Image			•	•			•	•					•	•

**Table 4:** Mapping of Domains and Themes to Trauma-informed Design (TID) principles.

	Reduce or Remove Adverse Stimuli	Reduce or Remove Environmental Stressors	Engage the Individual Actively in a Dynamic, Multi-Sensory Environment	Provide Ways for the Individual to Exhibit Their Self-Reliance	Provide and Promote Connectedness to the Natural World	Separate the Individual from others who may Be in Distress	Reinforce the Individual’s Sense of Personal Identity	Promote the Opportunity for Choice
**Safety and Security**								
Defensible environments								
Environmental stressors								
**Control**								
Self-reliance								
Territory								
**Enriched Environments**								
Connection to nature								
Environmental diversity								
Image								

## Supplementary Materials

The following supporting information can be downloaded at: https://www.mdpi.com/article/10.3390/ijerph192114279/s1, Table S1: Overview of content of reviewed literature in relation to domains and themes.
