# Supplementary material for: Trauma-Informed Design of Supported Housing: A Scoping Review through the Lens of Neuroscience
Source: Int J Environ Res Public Health. 2022 Nov 1;19(21):14279. doi: 10.3390/ijerph192114279 (PMC9658651; doi:10.3390/ijerph192114279)
Supplement: Supplementary file 1 [file ijerph-19-14279-s001.zip › ijerph-1896521-supplementary.pdf]

Table S1. Overview of content of reviewed literature in relation to domains and themes.

Reference	Safety and Security	Control	Enriched environments
Bollo & Donofrio, 2021 [12]	Defensible Environments:<br>• clear sightlines— preview space at thresholds and interior glazing (visibility)<br>• larger transitional spaces, multiple exits and wide doorways (escape)<br>Environmental Stressors:<br>• secure space for sleeping outside<br>• no fluorescent lights	Self-reliance:<br>• individually operated lighting<br>• 'teaching' kitchen<br>Territory:<br>• high ratio of common areas to private space and multiple/discrete spaces<br>• flexibility in furniture<br>• anti-institutional reception desks	Connection to Nature:<br>• visual and direct access to outdoor spaces, trees and planting<br>• preference for daylight<br>Environmental Diversity:<br>• varied lighting<br>• multiple common spaces with different sensory qualities<br>• opportunities for personalization (artwork/photos)
Datta, 2005 [66]	Defensible Environments:<br>• lines of sight from interior to exterior (visibility)<br>• architecture of the shelter 'blending in' (concealment)<br>• importance of secure external boundaries (secure boundaries)<br>Environmental Stressors:<br>• perception of 'bad neighborhood'	Self-reliance:<br>• convenience of location<br>• ability to prepare own food (access to kitchen and equipment) and importance of storage to keep things 'tidy and orderly'<br>• line of sight from laundry to children's play area<br>Territory:<br>• importance of private/personal space (bedrooms, bathrooms, kitchens)	Environmental Diversity:<br>• personalization (display of objects, moveable furniture)<br>Image:<br>• 'markers' of home (e.g. furniture, appliances, décor, size of space) relative to individual/context)
Huffman, 2018 [21]		Self-reliance:<br>• garden/food production promotes self-reliance, self-learning, and self-value<br>Territory:<br>• garden/food production as site of conflict (of control)	Connection to Nature:<br>• garden place of peace and quiet and promotes positive childhood memories<br>Image:<br>• 'grand' spaces promote positive feelings of being 'away' (hotel)

Koehn et al. 2020 [69]	Defensible Environments:<br>• social interaction only occurs in visible common areas (visibility)<br>• importance of personal spaces with lockable doors (secure boundaries)<br>Environmental Stressors:<br>• noise as trigger (avoid alarms and importance of acoustic separation)	Territory:<br>• importance of self-contained private spaces (bedrooms and bathrooms)<br>• use of common spaces limited due to institutional rules	
Lygum et al., 2019 [70]	Defensible Environments:<br>• not having to be uncertain as to what happens behind your back' (visibility)<br>• lines of sight to garden with good quality lighting (visibility)<br>• value of 'dark corners' as places to hide and visual screening for privacy (concealment)<br>• opportunity for courtyard typology to create secure boundaries in common outdoor spaces (secure boundaries)	Self-reliance:<br>• residents' desire for vegetable plot<br>Territory:<br>• importance of privacy within common spaces (visual screening and acoustic control)<br>• garden perceived as 'free space' (particular importance for children)<br>• uncertainty over access and use of common spaces— lack of clarity in visual cues and institutional 'rules'<br>• importance of multiple common spaces or defined areas to meet diverse needs	Connection to Nature:<br>• garden as therapeutic setting— direct immersion, connections with wildlife, food production and views of plants<br>Environmental Diversity:<br>• garden as opportunity for diverse sensory qualities (fruit, colors, smells)<br>• garden supports 'many ways to play' (diverse needs of children who have experience trauma)
McLane & Pable, 2020 [22]	Defensible Environments:<br>• importance of preview to common spaces (visibility)	Self-reliance:<br>• importance of cleanliness (of self and environment)<br>Territory:<br>• communal areas sized to support multiple activities (use distinct zoning with separation/screening).<br>• larger spaces with clearly delineated 'functional zones' with sense of privacy	Connection to Nature:<br>• Value of daylight and views of nature<br>Image:<br>• scarcity of furnishings results in 'overpoweringly institutional feeling'<br>• sense of hominess – cleanliness, acoustics, lighting quality, furniture, fixtures, accessories and décor

		• Use of common spaces influenced by spatial design factors— location in building, visibility into and from the space, wayfinding, size and adjacencies to kitchens and private rooms	• aesthetics found to be second most important factor in use of common spaces after location
McLeod & Walsh, 2014 [65]	Defensible Environments:<br>• peepholes, buzzers for entry (visibility)<br>• staff surveillance promotes sense of security (visibility)<br>• solid doors, locked facilities, locked personal storage (secure boundaries)<br>Environmental Stressors:<br>• perceived 'unsafe' and 'rundown' area contrasted with value of 'non-judgmental' communities and access to services	Territory:<br>• importance of designated private space (including bathrooms)	Image:<br>• creation of welcoming environment (shared areas) — cleanliness, décor, music, soft furnishings, flowers, books
Nuamah et al., 2021 [67]	Defensible Environments:<br>• awareness of surroundings— windows with views to exterior, no need to have 'back turned', no blind corners, preference for larger/open/circular spaces and reduced clutter (visibility)<br>• clarity in location of entrances and exits (wayfinding), wider walkways and hallways, free of obstacles (escape)<br>Environmental Stressors:		Connection to Nature:<br>• views of nature, feel of air (openable windows)<br>• integrate green space— plants and trees as calming environments<br>• preference for daylight<br>• color green as calming<br>Environmental Diversity:<br>• preference for 'vibrant' colors

	• unexpected noise as trigger and general concern about noise levels (need for good acoustic separation)<br>• odors as triggers and general concern over provision of natural ventilation/good air quality<br>• negative association of cramped spaces with combat		
Pable, 2012 [71]	Defensible Environments:<br>• importance of storage of possessions (for objects and children) (secure boundaries)<br>Environmental Stressors:<br>• 'sense of space' (without altering floor area) reduces feelings of claustrophobia and enhances children's behavior e.g. Dutch door (views out), storage (reduces visual clutter), mirror, visual separation (curtains)	Self-reliance:<br>• storage of possessions enhances perceptions of control (organization/cleanliness)—general importance of cleanliness of built environment)<br>Territory:<br>• importance of maintaining personal space/privacy in shared bedrooms<br>• clear boundaries for private/personal space (visual and physical separation) e.g. curtains, Dutch door (substantially enhanced perceptions of privacy and control) and 'markers' of personal territory (e.g. display boards) within shared family bedroom	Environmental Diversity:<br>• enhanced opportunities for occupation and activity through flexible furniture/décor (seating cubes, bolsters, cushions, rugs)<br>• elements as 'vehicles for imagination' (Dutch door as 'store', curtains as 'hide and seek')<br>• opportunities for personalization—integrated shelving, pin boards, external signage (thresholds)
Peters et al. 2020 [63]			Connection to Nature:<br>• direct access to nature (sensory experiences and interaction with living (animals) and 'non-living' (plants) associated with higher parental need satisfaction and lower

			parental need frustration, particularly for parents with young children
Peters et al., 2021 [64]			Connection to Nature:<br>• direct access to nature (sensory experiences and interaction with living (animals) and 'non-living' (plants) 'significantly affected basic psychological need fulfilment', but not overall wellbeing
Refuerzo & Verderber, 1989 [72]	Environmental Stressors:<br>• perceived sense of neighborhood safety is related to quality of sleep, relaxation and perception of ability to attain personal goals.	Territory:<br>• importance of personal space	Image:<br>• exterior appearance of the shelter, nearby buildings, and quality of children's outdoor play areas affects use<br>• neighborhood location and presence of trees and vegetation affect feelings of personal status<br>• quality of interior spaces including size, windows, views, amenities and overall appearance affect feelings of personal status<br>• use of the shelter is associated with size of spaces, quality of furnishings, daylight, lighting, and overall appearance
Refuerzo & Verderber, 1990 [68]	Defensible Environments:<br>• importance of windows with views to outside 'without sacrificing occupant safety' (visibility and concealment)<br>• screen building, entrances and on-site parking from street/public view	Self-reliance:<br>• importance of location in close proximity to shops and recreation facilities and provision of car parking<br>• visually connect play areas for children (surveillance)	Connection to Nature:<br>• landscaped 'outdoor rooms' and views to landscape create 'calming' settings<br>• benefits of access to daylight<br>Image:<br>• 'cheery' interior spaces

	with set-backs and use of trees and bushes (concealment)<br>Environmental Stressors:<br>• quiet site location<br>• smaller shelters perceived as more stressful	Territory:<br>• importance of personal space for each family and individual including storage of personal belongings<br>• flexible interior spaces support different uses	• neighborhood context and exterior appearance of the shelter important— 'attractive' neighborhood and 'well maintained' exterior
